# Midkine-Notch2 Pathway Mediates Excessive Proliferation of Airway Smooth Muscle Cells in Chronic Obstructive Lung Disease

**DOI:** 10.3389/fphar.2022.794952

**Published:** 2022-06-14

**Authors:** Tang Deng, Qifeng Huang, Kaiwen Lin, Jin Qian, Qi Li, Lihua Li, Shuangqin Xu, Hongfang Yun, Hangfei Wang, Xinxin Wu, Heng Liu, Guiyun Jin, Xiaoran Liu

**Affiliations:** ^1^ Department of Interventional radiology and vascular surgery, The First Affiliated Hospital of Hainan Medical University, Hainan Medical University, Haikou, China; ^2^ Key Laboratory of Emergency and Trauma of Hainan Medical University, Ministry of Education, Key Laboratory of Hainan Trauma and Disaster Rescue, Hainan Medical University, Haikou, China; ^3^ Hainan Women and Children’s Medical Center, Haikou, China

**Keywords:** Midkine-Notch2 pathway, inflammation, chronic obstructive lung disease, ASMC proliferation, airway remodeling

## Abstract

Inflammation-induced proliferation of airway smooth muscle cells (ASMCs) and subsequent airway remodeling is a hallmark of chronic obstructive lung disease (COPD). The role of midkine (MK) in COPD is unclear. In this work, we explored the role of MK-Notch2 signaling in COPD by inhibiting the expression of MK using lentivirus shRNA in ASMCs *in vitro* and instillation of AAV9-MK in the airway of a COPD rat model *in vivo*. The results demonstrated that LPS decreased ASMC migration and proliferation, increased apoptosis and induced the expression of MK and Notch2 signaling molecules. Inhibition of MK exacerbated the changes in migration and proliferation but decreased the expression of MK and Notch2 signaling molecules. Rats treated with smoke fumigation and LPS showed features of COPD. The small airways of COPD rats were remodeled and lung function was significantly reduced. The expressions of TGF-β, ICAM-1, HA, MMP-9, PC-III, and LN in BALF and the expression of MK and Notch2 signaling molecules were significantly increased in the COPD rats compared with controls. Inhibition of MK reversed these changes. In conclusion**,** the MK-Notch2 pathway plays a key role in airway remodeling induced by ASMC proliferation. Targeting the MK-Notch2 pathway may be a new strategy for improving airway remodeling and preventing progressive decline of pulmonary function in COPD.

## Introduction

Chronic obstructive pulmonary disease (COPD) is the third leading cause of death in the world, following myocardial infarction and stroke. With the increase in the number of smokers and the aggravation of air pollution, the incidence of COPD is predicted to continue to rise. The estimated annual death toll from COPD in 2050 is approximated at close to 6 million. At present, the number of COPD patients in China has exceeded 100 million, with a male to female ratio of approximately 2:1. The incidence rate among individuals over 60 years old is 27% (Spero et al., 2017; Disease, 2018) COPD is not only detrimental to the health, quality of life and longevity of the individual, but also poses a heavy burden on the family and society (Iheanacho et al., 2020)

Cigarette smoking is the primary cause of COPD. Harmful particles in smoke directly damage bronchial mucosa, cause chronic inflammation in the small airways and stimulate airway smooth muscle cell (ASMC) proliferation, leading to smooth muscle hypertrophy. Chronic inflammation weakens the immune defense of the respiratory system (Ritchie and Wedzicha, 2020). Macrophages that are recruited to the sites of inflammation release proteases that break down the airway epithelial basement membrane. Decomposition of extracellular matrix produces hyaluronic acid (HA), laminin (LN) and type III procollagen (PC-III), which are deposited in the airway to cause airway remodeling. Together, these processes lead to non-reversible airway remodeling and COPD (Hatipoğlu, 2018). Together, airway remodeling is a complex pathological process that consists of multiple factors and components interacting to cause structural changes in the airway resulting in progressive worsening of airflow limitation. Current treatment of airway remodeling is mostly centered on the release of airway smooth muscle spasm, airway dilation, anti-infection, and anti-inflammation. Overproliferation of ASMCs is also an important cause of airway remodeling, and targeted therapy for airway smooth muscle cell proliferation has rarely been reported (Chan et al., 2019).

Midkine (MK) is a novel heparin-binding growth factor discovered by Japanese researchers in the late 1980s (Kadomatsu et al., 1988). MK is a pleiotropic molecule that is involved in functions such as chemotaxis, mitosis, anti-apoptosis activity and carcinogenesis (Muramatsu, 2014; Sato and Sato, 2014). MK is highly expressed in many malignant tumors, including pancreatic cancer and neuroblastoma. Notch2 is one of the receptors for MK in humans (Kosugi et al., 2007). MK activates the Notch2 signaling pathway, up-regulates the downstream target HES1 and promotes tumor cell proliferation while inhibiting tumor cell apoptosis (Kishida et al., 2013) Notch signaling has been found to be upregulated in pulmonary fibrosis, and inhibition of Notch significantly alleviated pulmonary fibrosis. Our preliminary results showed that MK was highly expressed in a rat model of COPD. We speculate that the MK-Notch2 signaling pathway may promote inflammation and excessive proliferation of ASMCs, resulting in airway stenosis and the progressive decline of pulmonary function in patients with COPD. In this work, the role of MK-Notch2 signaling in COPD was explored by inhibiting the expression of MK using lentivirus shRNA in ASMCs *in vitro* and instillation of AAV9-MK in the airway of a COPD rat model *in vivo*. The study aims to provide new ideas for therapeutic strategies that have the potential of slowing down the progressive decline of pulmonary function in patients with COPD.

## Materials and Methods

### Cell Culture

Rat ASMCs (Shanghai Qingqi, China) were cultured, passaged and cryopreserved in a complete high-glucose medium supplemented with 10% fetal bovine serum (FBS) (BI, Israel). Cell experiments were conducted in a high-glucose medium supplemented with 3% FBS. In experiments, the ASMCs were divided into the following six groups: control, non-target shRNA, shRNA 413, control+LPS, non-target+LPS and shRNA 413+LPS. Lentivirus shRNA used to inhibit the expression of MK were designed and purchased from Jikai Gene, Shanghai, China. Successful transfection was observed under fluorescence microscope and verified by western blot and qPCR methods.

### Animal Model

Adult male SD rats, weighing 160 ± 10 g, were purchased from Tianqin, Hunan, China and housed in the Drug Safety Evaluation Center of Hainan Medical College. All animal experiments were carried out in accordance with the recommendations of the International guidelines for the care and use of laboratory animals. The experimental protocols were approved by the Animal Ethics Committee of Hainan Medical College. Rat model of COPD was established by exposure to LPS combined with smoking. LPS (200 µL) was instilled via tracheal intubation 10 min after anesthesia with sodium pentobarbital (40 μg/g) injected intraperitoneally. LPS was instilled at day 1, 10, 25, 40, 55, 70, and 85. Rats were exposed to cigarette smoke for 40–45 min each time via a homemade smoking box once in the morning and once in the evening for 100 days. The cigarette used for smoking had a nicotine content of 1.2 mg and tar content of 13 mg per cigarette. A total of 24 rats were divided into 4 groups with 6 rats in each group: control, COPD, COPD+AAV9-non-target and COPD+AAV9-MK. The control group received 300 μL of normal saline (NS) per rat. The COPD group was exposed to combined smoke fumigation and instillation of LPS for 100 days. The COPD group received 300 μL of normal saline (NS) per rat. The COPD + AAV9-non-target rats were instilled with 1.5 × 10^11^ vg/300 µL AAV9-non-target per rat. The COPD + AAV9-MK rats were instilled with 1.5 × 10^11^ vg/300 µL AAV9-MK per rat. The rats were sacrificed 21 days after AAV9 instillation. AAV9 were designed and purchased from Jikai Gene, Shanghai, China Table 1.

**TABLE 1:** Gene sequence of AAV9-MK-shRNA.

NO.	5′	STEM	Loop	STEM	3′
Midkine-shRNA (413)-a	ACCGG	CTG​AAG​AAG​GCT​CGG​TAC​AAT	TTCAAGAGA	ATT​GTA​CCG​AGC​CTT​CTT​CAG	TTTTT
Midkine-shRNA (413)-b	TCTAAAAAA	CTG​AAG​AAG​GCT​CGG​TAC​AAT	TCTCTTGAA	ATT​GTA​CCG​AGC​CTT​CTT​CAG	C

### Migration Assay

ASMCs were seeded in a 6-well plate, and the scratch test was performed by scratching the cell monolayer with a 200 μL pipette tip. Cell migration before and after treatment with 300 μg/ml LPS was observed under a light microscope. Images were taken at 0, 24, 48 and 72 h. The rates at which cells repopulate were analyzed by ImageJ software.

### Cell Proliferation and Apoptosis Assays

ASMCs were seeded in a 96-well plate and CCK-8 reagent (Dojindo, Japan) was added. They assay was performed according to the manufacturer’s instructions. The cell proliferation rate was calculated based on OD values. ASMCs were seeded in a 6-well plate and apoptosis was detected with an apoptosis detection kit (Shanghai Yisheng, China). The ASMCs were digested by EDTA-free trypsin and centrifuged. The supernatant was discarded. The cells were diluted to 3 × 10^6^ cells/ml. Six replicate tubes were divided into 6 groups. For each group, 100 µL of cell solution was added. The cells were centrifuged, and supernatant was discarded, 100 µL of Binding Buffer was added, 5 µL of Annexin V-FITC, 10 µL of PI Staining Solution, and then 400 µL of 1 × Binding Buffer was added. The solution was thoroughly mixed and FACS was used to detect the apoptosis rate of each group.

### Pulmonary Function Test

The rats were anesthetized by intraperitoneal injection of sodium pentobarbital (40 μg/g), tracheostomized and connected to a lung function instrument (Shanghai Tawang, China). The FEV mode was used to measure 0.3s FEV/FVC and maximal mid-expiratory flow (MMEF).

### ELISA

ELISA kits (Qingdao Baizhou, China) were used to quantify the levels of MK-Notch2 signaling molecules *in vitro*. Intracellular molecules were released from the cells by repeated freeze and thaw cycles. The cells were centrifuged and the supernatant was collected for quantification of MK, HES1, and Notch2 using ELISA. ELISA kits were also used to quantify the levels of inflammatory mediators transforming growth factor-β (TGF-β), intercellular adhesion molecule 1 (ICAM-1), HA, matrix metalloproteinase-9 (MMP-9), PC-III and LN in the bronchoalveolar lavage (BALF) of rats. Experiments and standard curves were performed according to the manufacturer’s instructions.

### qPCR

The mRNA levels of MK-Notch2 signaling molecules MK, Notch2 and HES1 were semi-quantified with qPCR. RNA extraction, reverse transcription and fluorescence quantification were carried out with kits purchased from Yisheng, Shanghai, China. Primers were designed by Shanghai Shenggong, China; the primer sequences are listed in Table 2. The PCR amplification program consisted of 40 cycles. Actin mRNA was used for the normalization of gene expression.

**TABLE 2:** Primer sequences.

Name primer sequence length		
ACTIN	F:GCCTTCCTTCCTGGGTATGG	186 bp
	R:TCCGATTCAACTCATACTGC	
Midkine	F:TTGCTACACCTAGTACCCAAAG	169 bp
	R:CTCGATAACAGGTATCAGGGTG	
HES1	F:GTAGGGGTCAGTGGCTTAGC	106 bp
	R:GAGGGTGGGGTAGGCTAAGA	
Notch2	F:TGCGAGACCAACATCAACGAGTG	128 bp
	R:TCAGGCAGAAGCAGAGGTAGGC	

### Hematoxylin and Eosin staining

Lung tissue from rats were fixed with 4% formaldehyde, dehydrated and embedded in paraffin. The paraffin blocks were sectioned into thin slices and stained with H&E (Biosharp, China) after deparaffination and rehydration.

### Immunofluorescence

Cryosectioned lung tissue was immersed in 4°C acetone and washed with PBS, and the cell membranes were permeabilized with 0.3% Triton X-100 (Biosharp, China). The slides were incubated with antibody against green florescent protein (GFP, 1:500, Abcam, UK) under room temperature and washed with PBS. The cell nuclei were visualized with DAPI (1:2000, Abcam, United Kingdom).

### Western Blot

Protein extraction was performed using RIPA lysis buffer (Wuhan Boster, China). Protein samples were separated by electrophoresis and transferred to polyvinylidene difluoride (PVDF) membranes. The membrane was blocked with 5% skimmed milk for 90 min at room temperature. The PVDF membrane was incubated with the primary antibody at 4 °C overnight on a shaker. The membrane was then incubated with secondary antibody at room temperature for 1 h. The membrane was placed into a chemiluminescence machine to visualize the protein bands after washing. ImageJ software was used to analyze band densities. Tubulin was used as a loading control. All antibodies were purchased from Abcam, UK; the antibody dilutions used were as follows: MK 1:1000; Notch2, 1:1000; HES1, 1:500.

### Immunohistochemistry

Lung tissue slices were deparaffinized and washed with PBS, immersed in citric acid for antigen retrieval and incubated with 3% H_2_O_2_. The slices were washed with PBS and blocked with 4% sheep serum before incubation with the primary antibody (same concentrations as for western blot) under room temperature. The slides were stained with DAB, counterstained with hematoxylin and dehydrated with increasing concentrations of alcohol and xylene before sealing.

### Data and Statistical Analysis

Results are expressed as mean ± standard deviation (x ± SD). Sample means were compared pairwise using one-way ANOVA. *p* < 0.05 was considered statistically significant. All statistical analysis was carried out by Graphpad software.

## Results

### IC_50_ of LPS in ASMCs and the Efficacy of Different Lentivirus shRNAs in Silencing MK

**FIGURE 1:**
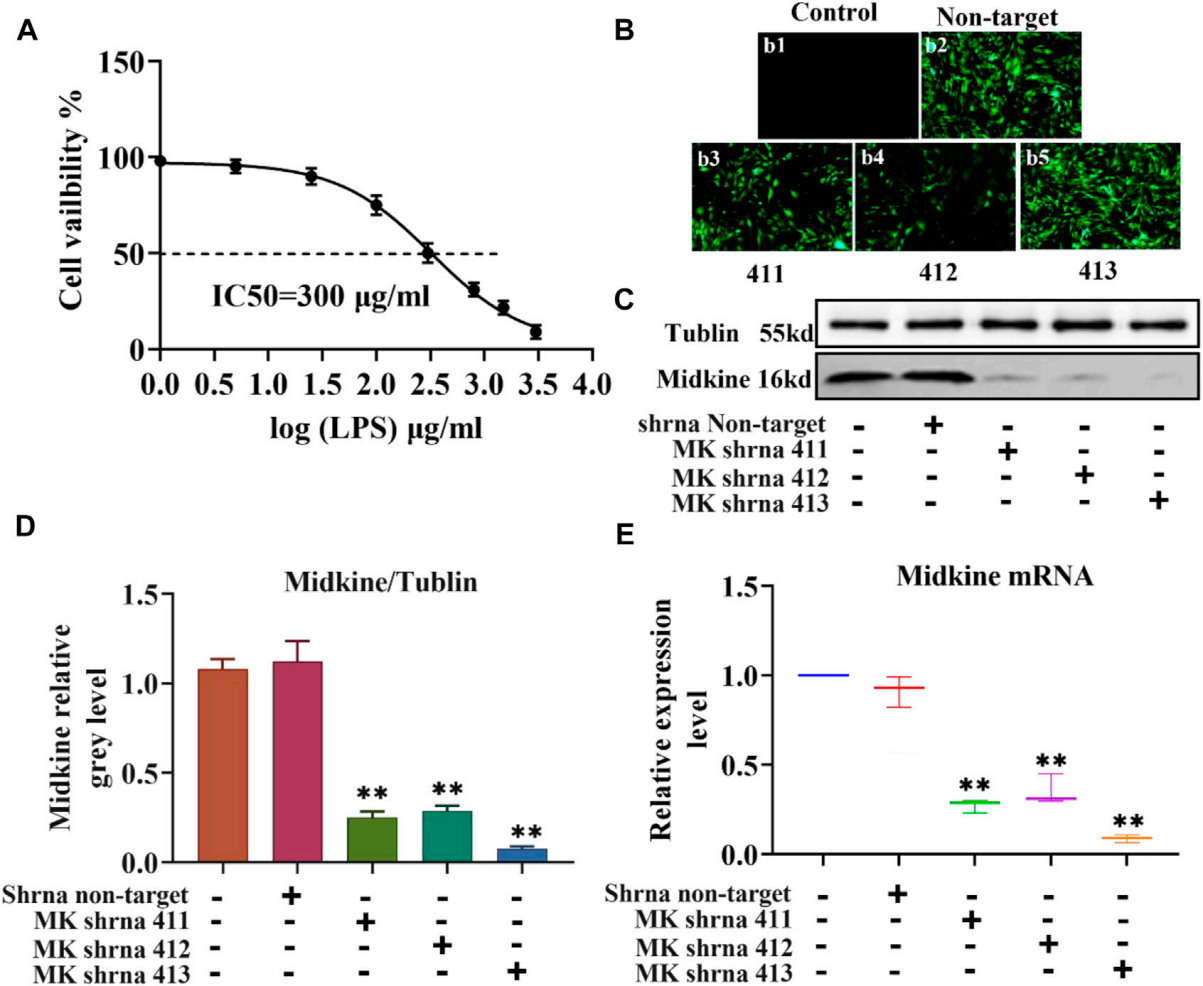
IC_50_ of LPS in ASMCs by LPS and the efficacy of different lentivirus shRNAs in silencing MK. **(A)** Dose-response curves for determining the IC_50_ of LPS in ASMCs. *n* = 5, x±SD. **(B)** The expression of different lentivirus in ASMCs (200x). **(C)** Western blot analysis of ASMCs transfected with the indicated lentivirus. **(D)** Quantification of the MK protein content relative to the tubulin loading control. **(E)** qPCR was performed on ASMCs transfected with the indicated lentivirus; the relative MK mRNA expression levels normalized to the control group are shown. * *p* < 0.05 and ** *p* < 0.01 compared with the control group.

The injury of ASCMs was first induced with LPS and the IC_50_ value of LPS was determined. ASMCs were stimulated with increasing concentrations of LPS (1 μg/ml, 5 μg/ml, 25 μg/ml, 100 μg/ml, 300 μg/ml, 800 μg/ml, 1500 μg/ml and 3000 μg/ml) and the effect of LPS on proliferation was detected using the CCK-8 kit. The results showed that increased concentration of LPS caused decreased cell viability and that the cell viability was close to zero at the highest LPS dose (3000 μg/ml) (Figure 1A). The IC_50_, i.e. the concentration of LPS that inhibits ASMC activity by 50%, was found to be 300 μg/ml. Then, we transfected ASCMs with different lentivirus shRNAs targeting MK and examined the expression of GFP under a fluorescence microscope at 72 h after transfection. There was no expression of GFP in the control group and strong fluorescence expression in the non-target group. Among the different shRNAs targeting MK, shRNA 411, shRNA 412 and shRNA 413 all resulted in different florescence intensities, and shRNA 413 displayed the strongest fluorescence signal (Figure 1B). We also performed western blot analysis on ASMCs transfected with lentivirus shRNAs. The expression level of MK mRNA was also quantified with qPCR. While there was no significant difference in MK protein (Figures 1C,D) or mRNA expression level (Figure 1E) between the non-target shRNA group and the control group, MK protein and mRNA expression levels in the shRNA 411, 412 and 413 groups were all significantly lower than that in the control group. MK levels were the lowest in the shRNA 413 group; the inhibition rate of MK protein was 91 and 93% for MK mRNA.

### Cell Migration, Proliferation and Apoptosis of ASMCs Before and After LPS Treatment and Inhibition of MK Expression

**FIGURE 2:**
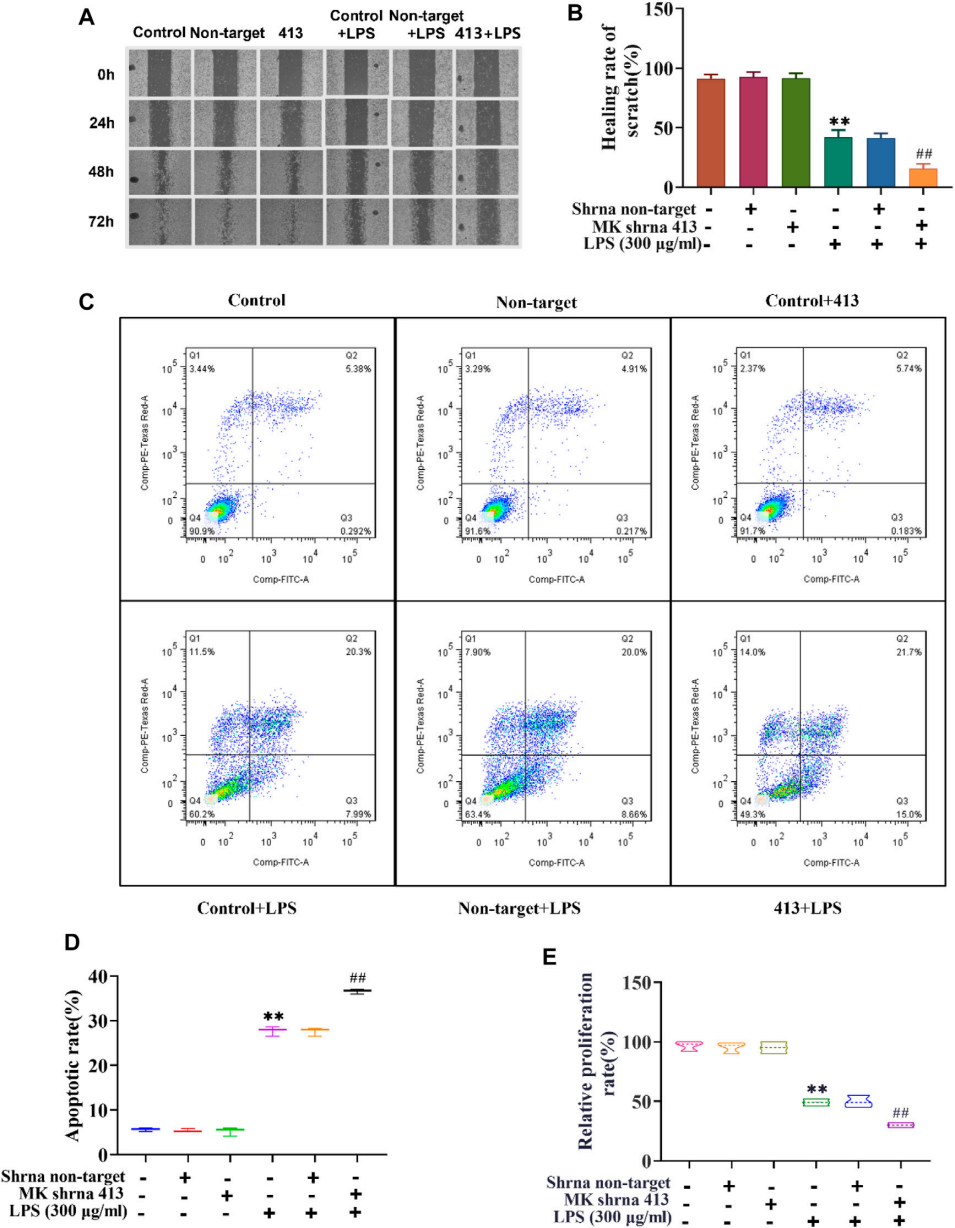
Cell migration, proliferation and apoptosis of ASMCs before and after LPS treatment and inhibition of MK expression. **(A)** Images taken at 0, 24, 48 and 72 h after the cell scratch. **(B)** The scratch healing rate of each group was calculated by ImageJ software after 72 h. **(C)** Flow cytometry analysis of cells in each group. **(D)** The apoptosis rate of each group was determined. **(E)** The cell proliferation rate was detected by CCK-8 assay. *n* = 3, x±3. * *p* < 0.05, ** *p* < 0.01 compared with the control group. # *p* < 0.05, ## *p* < 0.01 compared with control+LPS.

Next, we used scratch assay, flow cytometry and CCK-8 to explore the effect of MK on the migration, apoptosis and proliferation of ASMCs. As illustrated in Figure 2A, the results of the scratch test showed that there was no significant difference in the scratch healing rate between the non-target shRNA and control groups. The scratch healing rate of the shRNA 413 group (91.66%) was not significantly different from that of control group (91.33%) (Figure 2B). The scratch healing rate of control + LPS group (42%) was not significantly different from non-target + LPS group (41.33%), but the rate was significantly lower than that of the control group. The scratch healing rate of the shRNA 413+LPS group (15.66%) was significantly lower than that of the control + LPS group. The apoptosis rate of cells in each group was detected by flow cytometry after 72 h of treatment. The results showed that there was no significant change in apoptosis rate between the non-target (5.13%), control (5.67%) and shRNA 413 groups (5.92%) (Figures 2C,D). The apoptosis rate in the control + LPS group (28.66%) was significantly higher than that in the control group (5.67%). There was no significant difference in the apoptosis rate between the non-target + LPS (28.29%) and control + LPS groups (28.66%). The apoptosis rate in the shRNA 413+LPS group (36.7%) was significantly higher than that in the control + LPS group (28.66%). The proliferation rate of cells in each group was detected by CCK-8 assays after 72 h of treatment. The results showed that the proliferation rate of the non-target group (96.67%) and shRNA 413 group (95%) were not significantly different from that of the control group (95.33%) (Figure 2E). The proliferation rate of the control + LPS group (49%) was significantly lower than that of the control group. The proliferation rate of the non-target + LPS group (49.67%) was not significantly different from that of the control + LPS group. The proliferation rate of the shRNA 413+LPS group (30%) was significantly lower than that of the control + LPS group.

### Characterization of the MK-Notch2 Signaling Pathway *in vitro*

**FIGURE 3:**
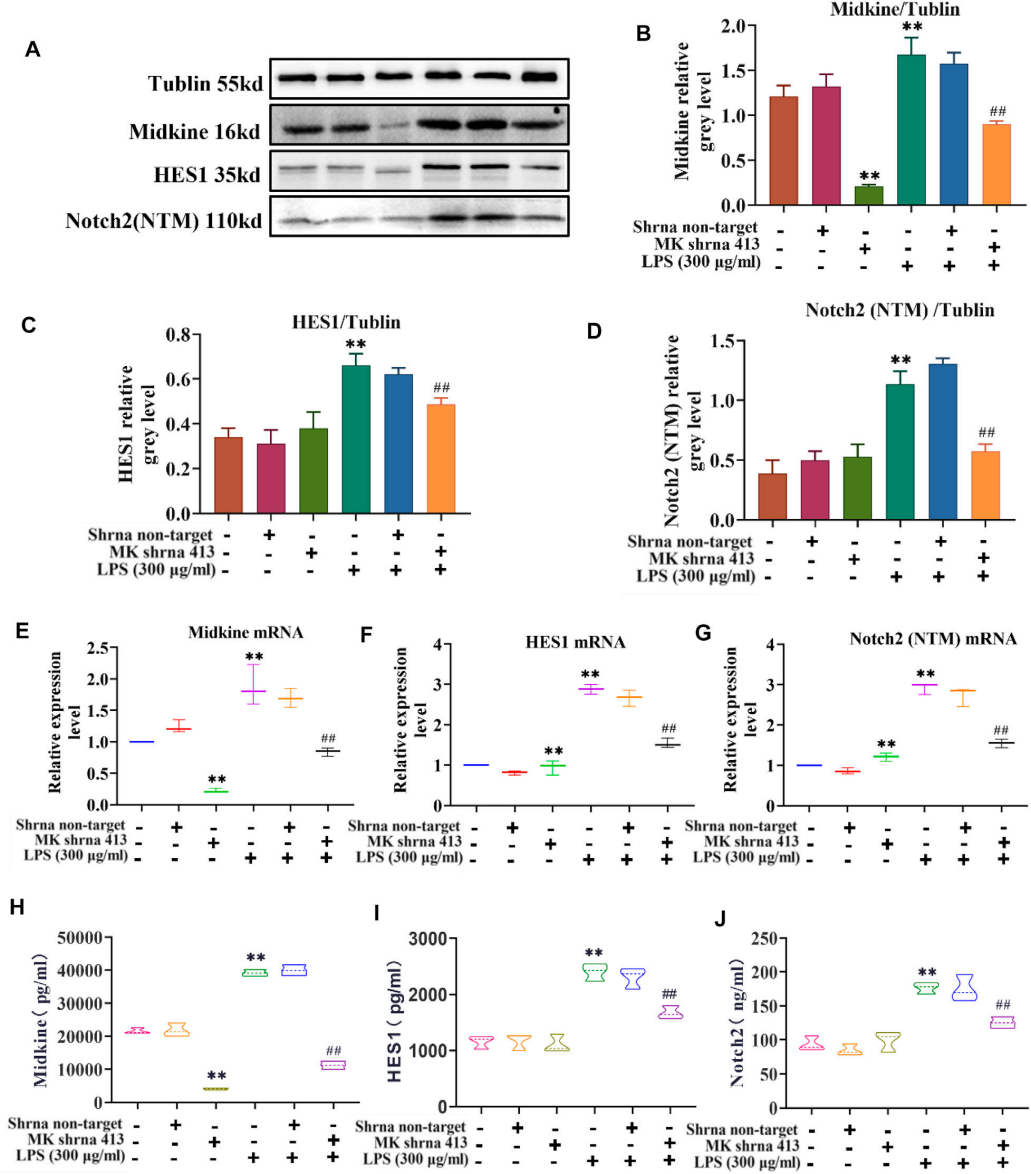
Characterization of the MK-Notch2 signaling pathway *in vitro*. **(A)** Western blot analysis. **(B)** Quantification of MK levels normalized to tubulin. **(C)** Quantification of HES1 levels normalized to tubulin. **(D)** Quantification of Notch2 levels normalized to tubulin. **(E)** The relative expression of MK mRNA. **(F)** The relative expression of HES1 mRNA. **(G)** The relative expression of Notch2 mRNA. **(H)** MK concentration detected by ELISA. **(I)** HES1 concentration detected by ELISA. **(J)** Notch2 concentration detected by ELISA.

The relationship between MK and Notch2 signaling pathway in the injury of ASMCs was investigated. As depicted in Figure 3, western blot analysis of MK protein, ELISA of MK in cell supernatants and qPCR of MK mRNA expression showed that there was no significant difference in MK protein and mRNA expression between the non-target group and control group. MK was significantly lower in the shRNA 413 group and significantly higher in the control + LPS compared with the control group. MK in the non-target + LPS group was not significantly different from that of the control + LPS group, but MK in the shRNA 413+LPS group was significantly lower than that of the control + LPS group (Figures 3A,B,E,H). ELISA of MK from the supernatant of cells showed a similar trend as the qPCR results (Figure 3H). The protein and mRNA expression patterns of HES1 and Notch2 were similar. There was no significant difference in HES1 and Notch2 expression between the non-target, shRNA 413 and control groups. HES1 and Notch2 were significantly higher in the control + LPS group than in the control group. HES1 and Notch2 were not significantly different in non-target + LPS group compared with the control + LPS group. Both HES1 and Notch2 expressions in the shRNA 413+LPS group were significantly lower than those in the control + LPS group (Figures 3C,D,F,G). The expressions of HES1 and Notch2 were also detected in the supernatant by ELISA after repeated freeze-thawing and centrifugation (Figures 3I,J). The trend of HES1 and Notch2 expression was the same as that observed in qPCR.

### Inhibition of MK Expression in COPD Rats Alleviated COPD-Induced Morphological Changes in the Lungs

**FIGURE 4:**
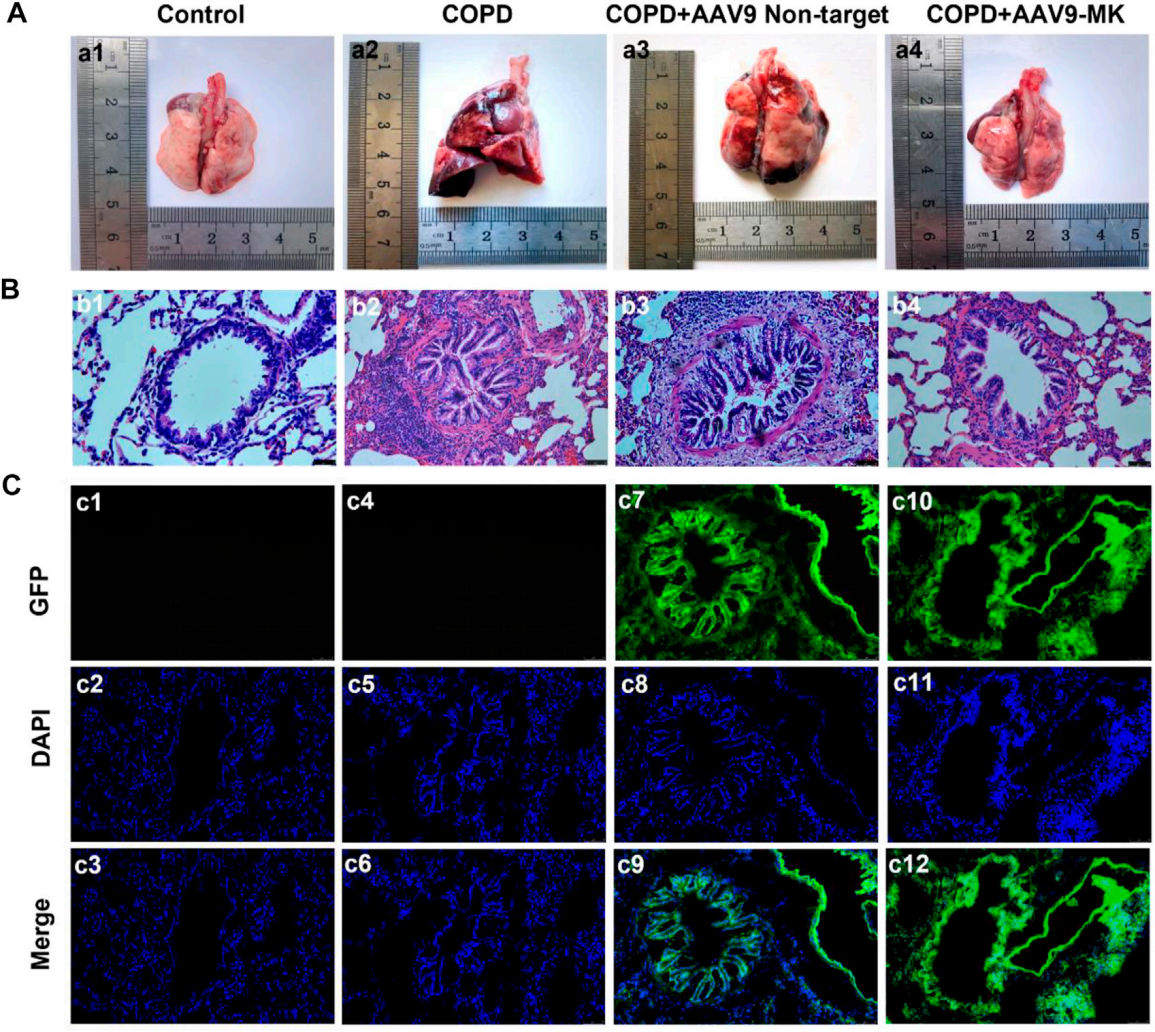
Morphology of lungs of COPD rats with or without inhibition of MK expression and the expression of GFP. **(A1–A4)** Representative photos of lung tissue. **(B1–B4)** H&E staining of lung tissue. **(C1–C12)** Fluorescence microscopy photos under GFP, DAPI and merge (synthesis) mode. Note: GFP expression is green, DAPI is blue (200x).

It was well known that inhibiting the proliferation of ASMCs could improve airway remodeling. Therefore, we would observe the changes in rat lung tissue and airway remodeling by inhibiting the expression of MK in COPD rat model. As shown in Figure 4a1, the surface of the lungs from rats in the control group was smooth, pinkish and without edema or nodule. Lungs of rats from the COPD and COPD + AAV9-non-target groups showed clear signs of inflammation and edema, with rough surface and congestion (Figures 4a2,a3). The signs of inflammation and edema were alleviated in the COPD + AAV9-MK group (Figure 4a4). H&E staining of lung tissue showed that the control group had no inflammatory cell infiltration. The small airway smooth muscle layer and lumen size were normal (Figure 4b1). The COPD and COPD + AAV9-non-target groups showed significantly increased small airway smooth muscle cells, airway muscle layer thickening, lumen deformation, stenosis, remodeling and peripheral inflammatory cell infiltration (Figures 4b2,b3). In the COPD + AAV9-MK group, the infiltration of inflammatory cells around the small airway was significantly reduced, the small airway smooth muscle cells decreased, the airway muscle layer became thinner, the lumen widened and the airway remodeling improved compared with the COPD + AAV9-non-target group (Figure 4b4). Cryosectioned lung tissue showed that the smooth muscle layer and lumen of the small airway in the control group were normal under DAPI mode and no green fluorescence was seen under GFP mode (Figures 4c1–c3). In the COPD group, the lumen was deformed, narrowed and remodeled under DAPI mode and no green fluorescence was seen under GFP mode (Figures 4c4–c6). In the COPD + AAV9-non-target group, the small airway lumen was deformed and narrowed, and the degree of remodeling was similar to that in COPD group under DAPI mode; green fluorescence was observed under GFP mode (Figures 4c7–c9). In the COPD + AAV9-MK group, the airway remodeling was improved and the lumen of small airway was widened; green fluorescence was observed under GFP mode (Figures 4c10–c12).

### Inhibition of MK Expression in COPD Rats Improves Lung Function and Decreases Inflammatory Mediators in BALF

**FIGURE 5:**
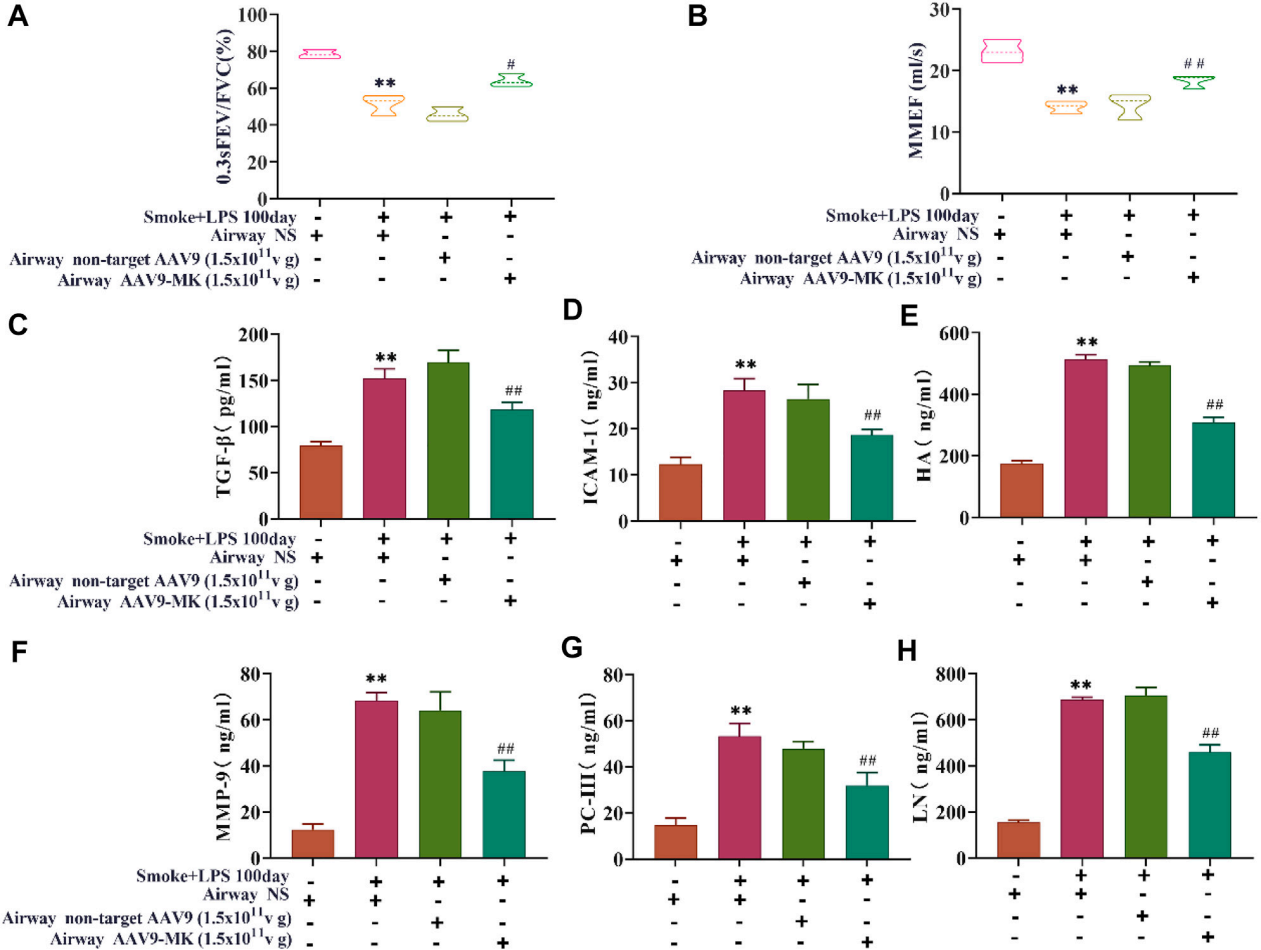
Pulmonary function and inflammatory mediators in BALF. **(A)** Pulmonary function parameter 0.3sFEV/FVC. **(B)** Pulmonary function parameter MMEF. **(C–H)** The concentrations of TGF-β, ICAM-1, HA, MMP-9, PC-III and LN in BALF. *n* = 6, x ± SD. * *p* < 0.05, ** *p* < 0.01 compared with the control group; # *p* < 0.05, ## *p* < 0.01 compared with the COPD group.

In COPD rats, the relationship between MK expression, inflammatory mediators in BALF and lung function was further explored. As illustrated in Figures 5A,B, the pulmonary function parameters 0.3sFEV/FVC and MMEF were measured using the FEV mode. The results showed that 0.3sFEV/FVC and MMEF in the COPD group were significantly lower than those in control group. There was no significant difference in 0.3sFEV/FVC between the COPD + AAV9-non-target group and COPD group. The COPD + AAV9-MK group had significantly improved 0.3sFEV/FVC and MMEF compared with the COPD group. The levels of TGF-β, ICAM-1, HA, MMP-9, PC-III, and LN were measured in BALF by ELISA (Figures 5C–H). The results showed that levels of TGF-β, ICAM-1, HA, MMP-9, PC-III and LN were significantly higher in the COPD group than the control group. These inflammatory mediators were not significantly different between the COPD + AAV9-non-target and COPD group, but were significantly lower in the COPD + AAV9-MK group compared with the COPD group.

### Characterization of the MK-Notch2 Signaling Pathway *in vivo*

**FIGURE 6:**
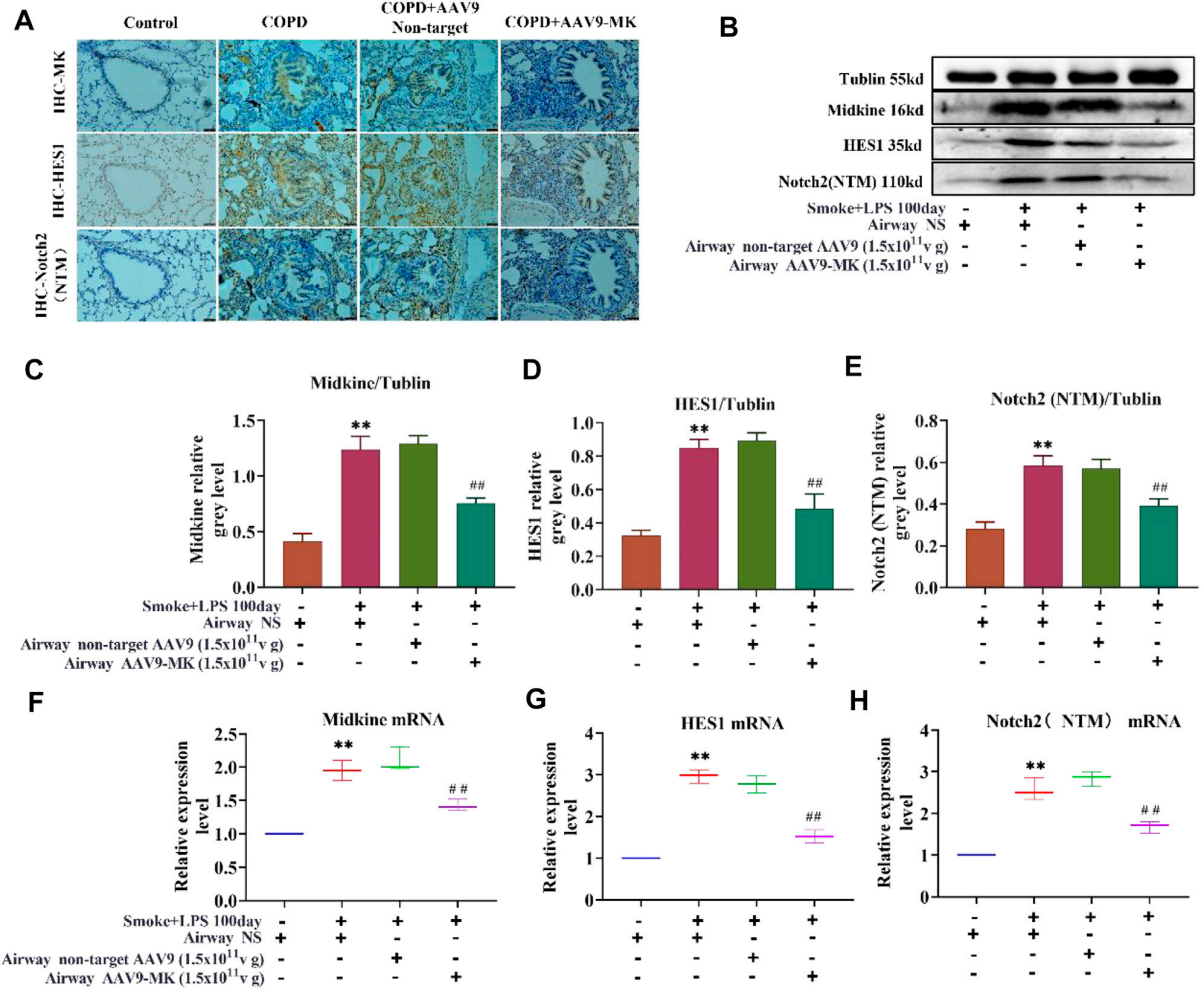
Characterization of the MK-Notch2 signaling pathway *in vivo*. **(A)** IHC of MK, HES1 and Notch2 protein in the lung tissue of the control group by IHC (200x). **(B)** Western blot analysis of MK, HES1 and Notch2 protein. **(C–E)** Quantification of MK, HES1 and Notch2 protein relative to tubulin. **(F–H)** Quantification of the relative expression of MK, HES1 and Notch2 mRNA in the lung tissue. * *p* < 0.05, ** *p* < 0.01 compared with the control group; # *p* < 0.05, ## *p* < 0.01 compared with the COPD group.

As depicted in Figure 6A, IHC of lung tissue showed that the expression of MK, HES1 and Notch2 was increased in COPD group compared with control. There was no significant difference in MK, HES1 and Notch2 expression between the COPD + AAV9-non-target and COPD group. The expression of MK, HES1 and Notch2 was significantly lower in the COPD + AAV9-MK group than that in the COPD group. A similar expression pattern was seen in the western blot analysis of protein extracted from lung tissue as well as the mRNA expressions of these signaling molecules by qPCR (Figures 6B–H).

## Discussion

MK is highly expressed in a variety of inflammatory diseases such as rheumatoid arthritis and Crohn’s disease (Shindo et al., 2017). MK is chemotactic and promotes the accumulation and infiltration of inflammatory mediators (Inoh et al., 2004; Zhang et al., 2015). MK also stimulates the release of macrophage inflammatory protein (MIP-2), which attracts and recruits macrophages and neutrophils. In the airway, MK-stimulated neutrophil recruitment aggravates the inflammatory response (Sato et al., 2002; Weckbach et al., 2014). Cigarette smoking is the most important risk factor for COPD. Harmful substances in smoke cause ASMC injury and stimulate the repair process, which lead to excessive proliferation of ASMCs. ASMC proliferation is closely related to the occurrence of COPD.

In our study, we induced injury of ASMCs with LPS and investigated the role of MK on proliferation, migration and apoptosis of injured ASMCs by inhibiting MK expression in ASMCs by lentiviral shRNA. Our results showed that inhibition of MK did not cause any changes in cell migration, proliferation and apoptosis in ASMCs that were not treated with LPS. Some studies have shown that the expression of MK is high in the embryo and decreases after birth, and the expression of MK in the kidney, small intestinal epithelium and other parts of the body is very low in healthy adult animals (Kadomatsu and Muramatsu, 2004). Inhibition of MK does not affect normal physiological function in healthy people. This is consistent with our finding that inhibition of MK expression in normal ASMCs had no effect on cell migration, proliferation and apoptosis. However, when the ASMCs were exposed to LPS, the expression of MK began to increase. The cells showed signs of acute injury, with decreased migration and increased apoptosis. When the expression of MK was inhibited in cells treated with LPS, ASMC viability and migration further decreased, and the apoptosis rate was higher. These results support the role of MK in cell proliferation, migration and inhibition of cell apoptosis in response to injury.

Research on COPD has been performed using a wide range of animal models (Wright and Churg, 2010; Polverino et al., 2015). Many animals have been used to establish COPD models, including mice, SD rats, goats, dogs and monkeys. However, rats and mice are considered the best choice because their genome has been sequenced in detail and the relatively low price associated with this model system. There is currently no national or international consensus on the evaluation criteria for COPD models in rats. In a review of the literature, 0.3sFEV/FVC, MMEF, histopathological signs on H&E staining and inflammatory factors in BALF were identified as common criteria (Ghorani et al., 2017). In this study, the COPD rats had a significantly lower 0.3sFEV/FVC and MMEF than control rats, suggesting airflow limitation and pulmonary function decline. The measured inflammatory mediators were all significantly increased in BALF, suggesting chronic inflammation in the airway. H&E staining showed neutrophil infiltration around the airway, airway smooth muscle thickening, deformation and stenosis of airway lumen, suggesting airway remodeling. Taken together, the results show that our rat COPD model reflects the pathophysiological changes of COPD.

Long-term smoke causes chronic airway inflammation in patients with COPD, stimulating airway mucosa to produce TGF-β and MMP-9, which leads to protease/anti-protease imbalance, alveoli destruction, hydrolyzed alveolar elastic fibers, alveolar fusion, and finally emphysema (Vlahos and Bozinovski, 2014). MMP-9 can directly damage the basement membrane of airway epithelium and cause breakdown of the extracellular matrix (ECM), releasing HA, LN and PC-III (Seemungal et al., 2008). Airway injury activates TGF-β and promotes the secretion of HA, LN and PC-III from the ECM (Mahmood et al., 2017; Di Stefano et al., 2018). HA, LN and PC-III deposit in the airways, leading to accelerated airway stenosis and remodeling (Westergren-Thorsson et al., 2018). Airway injury can also cause upregulated expression of ICAM-1 to promote the adhesion of neutrophils to airway epithelial cells, drive neutrophils to migrate into the airway, and thereby aggravate the inflammatory response (Zimmerman et al., 1994). Chronic airway inflammation accelerates the destruction of lung tissue and leads to airway remodeling, persistent airflow limitation, decreased lung function, and finally COPD. In our *in vivo* experiments, we also found that the expression of TGF-β, ICAM-1, HA, MMP-9, PC-III, and LN in BALF were significantly increased in COPD rats. Decreased pulmonary function and airway remodeling were also seen. Upon inhibition of MK expression in COPD rats with AAV9-MK, the lung function parameters 0.3sFEV/FVC and MMEF both increased, while the inflammatory mediators in BALF and neutrophil infiltration decreased; the airway smooth muscle layer also became thinner and the airway lumen widened. We also saw an improvement of airway remodeling in H&E-stained lung tissue sections. Taken together, the results suggest that MK plays an important role in COPD pathology and targeting the MK-related signaling pathways can reduce the inflammatory reaction, improve airway remodeling and enhance lung function.

MK expression is significantly increased in many malignant tumors, including gastric cancer, pancreatic cancer, lung cancer, breast cancer, colorectal cancer, esophageal cancer, hepatocellular carcinoma and bladder cancer (Ren and Zhang, 2006; Krzystek-Korpacka et al., 2012; Zhao et al., 2012; Yao et al., 2014; Li et al., 2015; Yuan et al., 2015; Song et al., 2016; Vu Van et al., 2016). MK has been shown to promote tumor cell proliferation and inhibit tumor cell apoptosis. The specific inhibitor of MK APT-1 significantly reduced the volume and weight of neuroblastoma and also reduced the expression of the nuclear transcription factor HES1 in the Notch2 signaling pathway. The HES1 transcription factor promotes downstream cell proliferation and inhibits apoptosis. The Notch2 signaling pathway has also been shown to be involved in the chemotactic inflammatory response (Danahay et al., 2015; Zhang et al., 2017; Carrer et al., 2020). In patients with asthma, inhibiting the Notch signaling pathway in goblet cells reduces inflammation and metaplasia and improves airway remodeling. MK promotes epithelial-mesenchymal transition (EMT), and inhibiting both MK and Notch signaling pathways alleviates pulmonary fibrosis. These studies suggest that the MK-Notch2 signaling pathway plays an important role in cell proliferation, inflammation and remodeling, which is consistent with the results of this study.

In the *in vitro* experiments, LPS was used to establish an acute injury model of ASMCs, and lentivirus shRNA was used to inhibit the expression of MK in ASMCs. We measured changes in MK and Notch2 signaling pathway factors (MK, Notch2 and HES1) and found that the MK-Notch2 signaling pathway in ASMCs was activated following acute injury. Inhibition of MK expression weakened the expression of Notch2 signaling pathway–related factors, as well as decreased ASMC proliferation and migration while increasing apoptosis. These results are consistent with findings from our *in vivo* experiments. In the COPD rat model, both MK and Notch2 signaling pathways were highly expressed. After inhibiting MK by AAV9, the expression of Notch2 signaling pathway factors also decreased. The NTM transmembrane protein is a factor in the Notch2 signaling pathway and is responsible for signal transduction; HES1 is a transcription factor downstream in the Notch2 signaling pathway that promotes cell proliferation and inhibits cell apoptosis. Thus, MK may enhance the signal transduction of the Notch2 signaling pathway through upregulation of the expression of the Notch2 receptor, the transmembrane protein NTM and downstream target HES1, and thereby regulate the cell cycle of AMSCs, promote ASMC proliferation, inhibit apoptosis, and lead to airway narrowing, thereby play a role in the development of COPD.

In summary, our findings showed that inhibiting the expression of MK *in vitro* and *in vivo* reduced the proliferation of ASMCs, decreased pulmonary inflammation and improved lung function and remodeling. Targeting MK, for example, by gene therapy, may be a new strategy to improve airway remodeling and prevent the progressive decline of pulmonary function in COPD.

## Data Availability

The original contributions presented in the study are included in the article/supplementary material, further inquiries can be directed to the corresponding authors.
